# Apoferritin/Vandetanib Association Is Long-Term Stable But Does Not Improve Pharmacological Properties of Vandetanib

**DOI:** 10.3390/ijms22084250

**Published:** 2021-04-20

**Authors:** Kateřina Jáklová, Tereza Feglarová, Simona Rex, Zbyněk Heger, Tomáš Eckschlager, Jan Hraběta, Petr Hodek, Matúš Kolárik, Radek Indra

**Affiliations:** 1 Department of Biochemistry, Faculty of Science, Charles University, Albertov 6, 128 00 Prague 2, Czech Republic; jaklovak@hotmail.com (K.J.); terezcern@gmail.com (T.F.); petr.hodek@natur.cuni.cz (P.H.); matus.kolarik@natur.cuni.cz (M.K.); 2 Department of Chemistry and Biochemistry, Mendel University in Brno, Zemedelska 1, 613 00 Brno, Czech Republic; simona1rex@gmail.com (S.R.); zbynek.heger@mendelu.cz (Z.H.); 3 Central European Institute of Technology, Brno University of Technology, Purkynova 656/123, 612 00 Brno, Czech Republic; 4 Department of Pediatric Hematology and Oncology, 2nd Faculty of Medicine, Charles University and University Hospital Motol, V Uvalu 84/1, 150 06 Prague 5, Czech Republic; Tomas.Eckschlager@fnmotol.cz (T.E.); janhrabeta@gmail.com (J.H.)

**Keywords:** vandetanib, apoferritin, neuroblastoma, medullary thyroid cancer, cancer targeting

## Abstract

A tyrosine kinase inhibitor, vandetanib (Van), is an anticancer drug affecting the signaling of VEGFR, EGFR and RET protooncogenes. Van is primarily used for the treatment of advanced or metastatic medullary thyroid cancer; however, its usage is significantly limited by side effects, particularly cardiotoxicity. One approach to minimize them is the encapsulation or binding of Van in- or onto a suitable carrier, allowing targeted delivery to tumor tissue. Herein, we constructed a nanocarrier based on apoferritin associated with Van (ApoVan). Based on the characteristics obtained by analyzing the average size, the surface ζ-potential and the polydispersive index, ApoVan nanoparticles exhibit long-term stability and maintain their morphology. Experiments have shown that ApoVan complex is relatively stable during storage. It was found that Van is gradually released from its ApoVan form into the neutral environment (pH 7.4) as well as into the acidic environment (pH 6.5). The effect of free Van and ApoVan on neuroblastoma and medullary thyroid carcinoma cell lines revealed that both forms were toxic in both used cell lines, and minimal differences between ApoVan and Van were observed. Thus, we assume that Van might not be encapsulated into the cavity of apoferritin, but instead only binds to its surface.

## 1. Introduction

**Figure 1:**
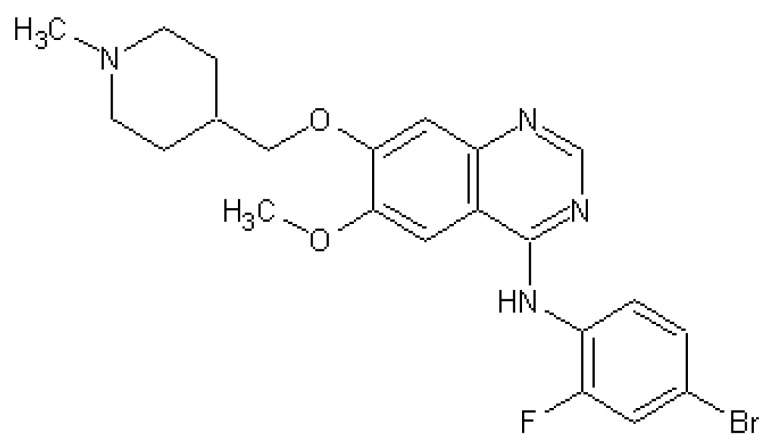
Structure of vandetanib.

Vandetanib (Figure 1, Van, Caprelsa^TM^, AstraZeneca, Cambridge, UK) is an oral anilinquinazoline compound with a molecular weight of 475 Da approved for the treatment of patients with unresectable symptomatic or metastatic medullary thyroid cancer [1]. Van is a tyrosine kinase inhibitor (TKI) with an affinity to multiple growth factor receptors, including VEGFR-2 (IC_50_ 40 nM), VEGFR-3 (IC_50_ 110 nM), EGFR (IC_50_ 500 nM), and the rearranged during transfection (RET) protooncogene (IC_50_ 130 nM) [2]. In addition, Van exhibits activity against protein tyrosine kinase 6 (BRK), tyrosine kinase with immunoglobulin and EGF domains-2 (TIE2), members of the ephrin (EPH) receptor kinase family and the Src family tyrosine kinases [3]. Therefore, Van is considered a multiple TKI. *N*-desmethyl vandetanib and vandetanib *N*-oxide have been identified as the two major oxidation products of Van in the rat, rabbit, and mouse hepatic microsomal systems, as well as in the human one. The formation of *N*-desmethyl vandetanib is mediated by cytochromes P450 (CYPs), especially CYP3A4 stimulated by cytochrome b5. Vandetanib *N*-oxide is formed by kidney and liver flavin-containing monooxygenase FMO1 and FMO3 [4]. The knowledge of these metabolic pathways is important as *N*-desmethyl vandetanib is of similar potency to Van, while vandetanib *N*-oxide exhibits 50-fold less activity than the parental drug [5]. Significant pharmacokinetic drug–drug interactions could lead to alterations in the plasma concentrations of Van, potentially resulting in a reduction in its efficacy or an increase in drug-related toxicity. Although Van is considered as the drug for targeted therapy and is usually well-tolerated, it also acts systemically. Therefore, the use of Van is connected with several side effects. The most common side effects are diarrhea, hypertension, fatigue, and nausea [6,7]. In addition, severe skin photosensitivity problems (including eczematous dermatitis, skin hyperpigmentation, acneiform lesions, delayed wound healing) have been reported during Van therapy. The life-threatening side effect is cardiotoxicity which is most often manifested by prolonged QT interval [8,9].

One way to minimize side effects which are caused by anticancer drugs is to use nanocarriers. This approach has been extensively investigated in the past few decades as it has shown to be promising in the area of drug delivery [10]. There are a variety of currently used nanoparticles (NPs) in research, including liposomes, polymeric NPs, micelles, viral NPs, metallic NPs or protein-based NPs [11]. However, despite extensive research on NP systems, there are only a few NP drug delivery systems, such as liposomes, albumin or polymeric micelles, that were approved by the U.S. Federal Drug Administration and European Medicines Agency for cancer treatment and for effective delivery of anticancer drugs into tumors [12]. NPs can target the tumor by passive and active targeting. Passive targeting is enabled by enhanced permeability and the retention (EPR) effect caused by leaky fenestration of tumor capillaries and a poor lymphatic drainage system [13,14]. Active targeting is achieved through surface modification of NPs with specific ligands, which have the ability of binding to specific receptors exclusively over-expressed by either tumor cells or tumor vessels [15].

Apoferritin is an apo-form of ferritin, a natural iron-storage protein, which has the appropriate properties to be a suitable drug-nanocarrier. It consists of 24 subunits that assemble into a hollow spherical structure, creating a protein cage with an internal cavity. Inside the cavity, up to 4500 iron ions can be stored [16,17]. The native ferritins are composed of two types of subunits: H-type (21 kDa) and L-type (19 kDa) that are encoded by separate genes with nonexchangeable functions. Every subunit is formed by an individual molecule that joins its neighboring subunit through noncovalent interactions [18,19]. The structure of apoferritin is quite stable. It is able to withstand biologically extreme temperatures (up to 70 °C) and a wide pH range (pH 2–10) for an appreciable period of time [20,21]. Apoferritin has a unique self-assembling ability, which is widely used by researchers in the field of nanomedicine. If ferritins are artificially expressed in an iron-free environment, apoferritin is formed. The empty inner cavity can be loaded with inorganic or organic substances [22]. It was shown that while disassembled, Apo protein subunits can be mixed with drug molecules which are encapsulated into the Apo cavity once reassembled [19,21,23]. The protein cage can be dissociated at a low pH (bellow 3), and conversely, the subunits can be re-associated at higher pH (pH 7–9) [24]. Another way to dissociate the protein structure is with the use of denaturing agents [25]. The binding of human ferritin to cell receptors is mediated through its H subunits, which interact with transferrin receptor 1 (TfR1) followed by endocytosis. This interaction and subsequent endocytosis are exploited in the usage of apoferritin for the targeted delivery of drugs to tumor cells, which increasingly express the TfR1 [25,26]. Other ferritin receptors are more prevalent in mice, including T-cell immunoglobulin and mucin domain protein 2 (TIM-2) and scavenger receptor class A member 5 (SCARA5) which bind ferritin through H and L subunits, respectively [27].

Thus, the aim of this study was to construct a nanocarrier based on apoferritin-containing encapsulated Van (ApoVan). The prepared Van-bearing nanocarriers were characterized and their biochemical and cytotoxic properties investigated.

## 2. Results

### 2.1. Preparation and Characterization of ApoVan

**Figure 2:**
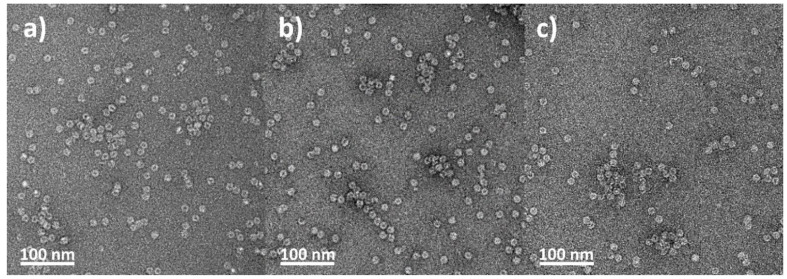
TEM visualization of: (**a**) pure apoferritin; (**b**) the freshly prepared ApoVan; (**c**) the ApoVan stored at 4 °C for a week.

We utilized reversible dissociation–association of apoferritin to prepare ApoVan. The visualization of apoferritin and ApoVan (Figure 2) and their size was determined using transmission electron microscopy (TEM) and quasielastic dynamic light scattering (DLS). The average size of apoferritin, ApoVan and ApoVan stored at 4 °C for one week (ApoVan^S^) measured by DLS did not differ (Table 1). The polydispersity index and the surface ζ-potential of prepared NPs were also determined using DLS (Table 1). The polydispersity index is used as a measure of the breadth of molecular weight distribution of polymers [28]. The surface ζ-potential shows the electrostatic potential at the electrical double layer surrounding NPs in the solution [29]. The average values of surface ζ-potential were negative for all samples (Table 1).

**Figure 3:**
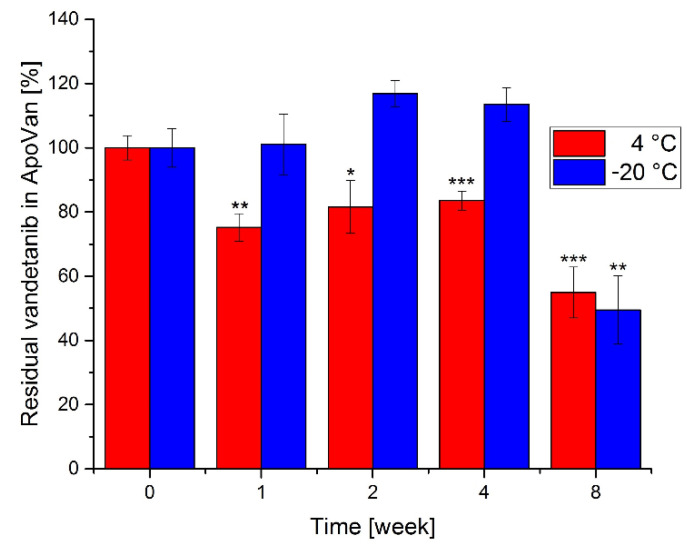
The stability of ApoVan at 4 °C and −20 °C. Values are mean ± SD from three independent experiments. * *p* < 0.05, ** *p* < 0.01, *** *p* < 0.001, significance was related to control values.

Next, we evaluated the stability of ApoVan at two different temperatures (4 °C and −20 °C) for up to 8 weeks. Compared to the amount of trapped Van in ApoVan at the time of preparation (week 0), the only significant release of Van from ApoVan (Figure 3) is visible after eight weeks of its storage at −20 °C. Although storage at 4 °C exhibits significant release even after one week, less than 20% is released after four weeks.

### 2.2. Release of Van from ApoVan Nanocarrier

**Figure 4:**
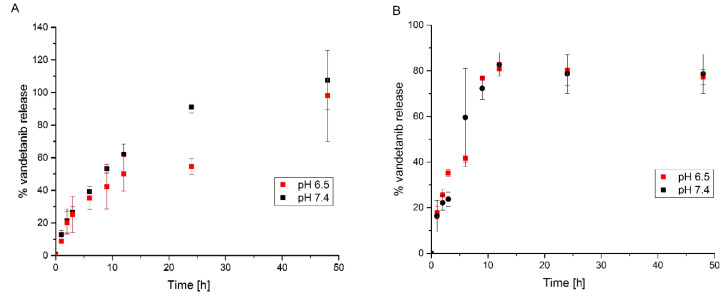
The kinetics of Van release from: (**A**) ApoVan; (**B**) ApoVan prepared without using reversible dissociation of apoferritin at pH 6.5 and 7.4 at 37 °C. Values are mean ± SD from three independent experiments.

Due to the Warburg effect or hypoxic conditions, the pH of tumor cells and their microenvironment is more acidic compared to the physiological pH of healthy tissues [30]. Thus, the release of free Van from ApoVan was monitored at two pH levels (pH 6.5 as a model of a cancer microenvironment, and pH 7.4 as the equivalent of healthy tissues). As shown in Figure 4A, the release of Van from ApoVan into pH 6.5 is almost indistinguishable from that at pH 7.4. There is a clear trend, which shows that during 48 h of incubation the whole amount of Van is released from apoferritin at pH 6.5 98% ± 30%, at pH 7.4 107% ± 20%. Therefore, we assume that encapsulation of Van molecules into the inner cavity of apoferritin is not efficient during the process of ApoVan preparation and that Van molecules probably only bind to the surface of the protein. To test this hypothesis, the same experiment was performed with ApoVan prepared without using reversible dissociation of apoferritin. The results show (Figure 4B) an almost identical trend as in the first experiment, even though no reversible dissociation of apoferritin was used in the preparation process. During 48 h of incubation, 77% ± 3% of Van was released at pH 6.5 and 79% ± 9% at pH 7.4. Thus, Van is gradually released into the acidic environment (6.5) as well as into the physiological (7.4) pH and the preparation process of ApoVan has little or even no effect on it.

### 2.3. Cytotoxicity of Free Van and ApoVan Nanoparticle in Neuroblastoma and Thyroid Carcinoma Cells

**Figure 5:**
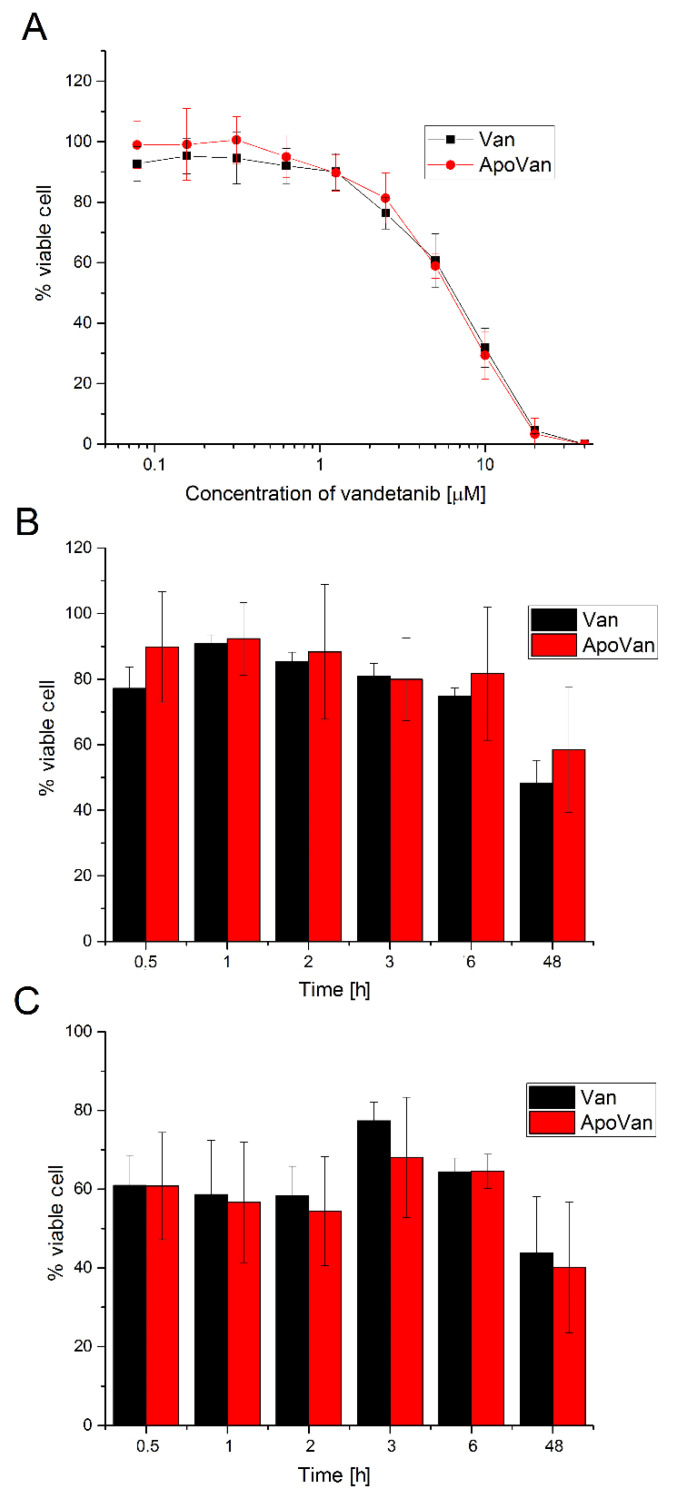
(**A**) The concentration dependence of free Van and ApoVan cytotoxicity in neuroblastoma cells UKF-NB-4 for 48 h. Values are mean ± SD from three independent experiments; (**B**) the time dependence of free Van and ApoVan cytotoxicity in neuroblastoma cells UKF-NB-4 for up to 48 h; (**C**) the time dependence of free Van and ApoVan cytotoxicity in thyroid carcinoma cells (TT) for up to 48 h. Values are mean ± SD from four independent experiments.

Although encapsulation of Van was not confirmed, Van was likely associated with apoferritin and, therefore, its effect on cell lines was tested. The cytotoxicity of free Van and ApoVan in the neuroblastoma cell line UKF-NB-4 and medullary thyroid carcinoma cell line (TT) was studied. UKF-NB-4 cell line was selected because of the TfR1 [31] and SCARA5 overexpression [32]. TT cell line was selected because Van is primarily used to treat advanced medullary thyroid cancer. The impact of different concentrations of Van on the viability of UKF-NB-4 cells being exposed to both forms of inhibitor (free Van, ApoVan) for 48 h is shown in Figure 5A. An apoptosis was induced in UKF-NB-4 cells already after the treatment with 0.625 μM free Van and ApoVan. With the increasing concentration of Van, the cell viability rapidly decreases, regardless of the Van form. However, it is obvious that there is almost no difference in the effect of free Van and ApoVan on the cytotoxicity in UKF-NB-4 cells. As there was no difference between free Van and ApoVan, other experiments were realized. UKF-NB-4 cells and TT cells were incubated with free Van (10 μM) and ApoVan (10 μM) for a different time period. It is evident that a significant decrease in UKF-NB-4 cell viability (Figure 5B) is noticeable already after 0.5 h of the treatment with free Van. In the case of ApoVan, a significant decrease occurs after 3 h. Results of the cytotoxicity test of both forms of Van with thyroid carcinoma cells (Figure 5C) should be considered from two viewpoints—the first, when the cell viability decreases from 0.5 to 2 h after the cell exposure to Van and ApoVan, and the second, when the cell viability first increases up to 70%, and then decreases again with the increasing exposure to the Van. The cytotoxic effect of both forms of inhibitor (Van and ApoVan) on both cell lines is slightly different. In general, the cytotoxicity of free vandetanib, as well as ApoVan, is slightly higher in medullary thyroid carcinoma cells than in neuroblastoma cells. However, all three experiments show almost no difference in the cytotoxicity between both forms of Van in the cell lines used.

## 3. Discussion

The knowledge of size and shape are essential parameters influencing NPs biodistribution in vivo and impact on the mode of cellular internalization. Rigid, spherical particles with the size of 10–100 nm have the highest potential for EPR effect while avoiding extravasation from normal blood vessels [33]. Even though there was no visible difference among apoferritin, ApoVan and ApoVan^S^ using TEM, there was a slight difference, although not significant among the average sizes measured by DLS (the average size of ApoVan was slightly smaller compared to the other two forms of apoferritin). The reasons for these small differences among NPs still need to be explained. One can speculate that they can result from the process of encapsulation. Specifically, some Apo subunits seem not to be properly reassembled and, thus, remain in solution. One-week storage at 4 °C increases the possibility of reassembling of all 24 subunits creating apoferritin structure. Therefore, the average size of ApoVan^S^ is comparable with apoferritin. Values of the polydispersive index indicate that pure apoferritin is more homogeneous than apoferritin associated with Van. However, there are differences between ApoVan and ApoVan^S^. Freshly prepared ApoVan is the least homogeneous. Thus, these results support the hypothesis that some time is needed to reassemble the apoferritin structure. The obtained values of surface ζ-potential were negative for all samples. High values of ζ-potential result in strong repulse moments among the particles, leading to the stability of these colloidal disperse systems. Together these results indicate that apoferritin is a suitable nanocarrier for Van loading.

Furthermore, the stability of ApoVan was monitored in time. As shown in Figure 3, ApoVan samples are stable during the storage at −20 °C for up to 4 weeks. Interestingly, all ApoVan samples exhibit a significant release of Van at 4 °C. However, the release might not be caused by the time instability of ApoVan. ApoVan samples are washed and stored in ultra-pure water, which has a lower pH than the environment before the ApoVan wash during preparation. A lower pH might cause the partial release of Van, because the apoferritin association is pH dependent. The fact that the concentration of Van after one-week of storage is like the concentration after four weeks supports this assumption.

On the base of the self-assembling ability of apoferritin, it was expected that, similar to encapsulated ellipticine [4] or doxorubicin [25], Van will be released from the ApoVan only when the pH value is decreased. This means that ApoVan might be stable and a negligible amount of Van will be released within 48 h of incubation at pH 7.4. In contrast, a larger amount of Van was expected to be released at pH 6.5 compared to pH 7.4. However, as it is shown in Figure 4A, almost the entire amount of Van was released from ApoVan during 48 h of incubation, regardless the pH used. These results indicate that Van is not completely encapsulated in the inner cavity of apoferritin, but most likely associated with the protein surface. To test this assumption, ApoVan samples prepared without using reversible dissociation of apoferritin and, therefore, without encapsulation of Van into the cavity, were prepared for comparison and the experiment was repeated. The trend of Van release from ApoVan prepared without using reversible dissociation of apoferritin (Figure 4B) was like that observed earlier with ApoVan (Figure 4A). Thus, we assume that during the preparation of ApoVan, Van does not encapsulate into the inner apoferritin cavity or diffuse very quickly through the pores in the apoferritin structure. Such a finding is not solely limited to Van. The inappropriateness of apoferritin to encapsulate another TKI, namely lenvatinib, was described recently [34]. Lenvatinib molecules are rather neutral during encapsulation and thus they are not effectively drawn into the inner cavity. Even in the case of protonated Lenvatinib, very slow and rare encapsulation was found by molecular dynamics simulations [34]. Due to poor water solubility and lenvatinib precipitation, these results indicate an inappropriateness of apoferritin to encapsulate lenvatinib. On the contrary, Van should be protonated during the encapsulation process [35,36]. The positive charge of the majority of Van molecules was confirmed in our laboratory during metabolic experiments [37]. Thus, the inefficiency of Van encapsulation might have the following explanations. Firstly, the process of drawing into the inner cavity is too slow. However, as Van is poorly soluble in water and no precipitation was observed, this explanation seems less realistic. Secondly, Van is bound to the outer surface of apoferritin. The net charge of apoferritin’s exterior surface is close to zero or slightly positive at pH 7.0 [38]. Although Van bears a positive charge, it may still interact with negatively charged or neutral residues on the surface. The changes in ApoVan ζ-potential compared with empty apoferritin strongly support this hypothesis. The third possibility is a free diffusion of Van into and out of the inner cavity. 

Although the encapsulation of Van into the internal cavity of apoferritin was not demonstrated, the effect of free Van and ApoVan on neuroblastoma and medullary thyroid carcinoma cells was examined. Due to the specific interactions of apoferritin with tumor cells, the cytotoxicity of ApoVan was expected to differ from the free Van. We expected a faster transport of Van molecules associated only with the surface of apoferritin into cells, with no necessity of apoferritin dissociation to release the inhibitor. Therefore, an even more extensive cytotoxicity of ApoVan was expected. Firstly, the concentration dependence of the free Van and ApoVan on the treatment of cells was examined. As shown in Figure 5A, the cell death was induced in UKF-NB-4 cells already after their treatment with 0.625 μM Van and ApoVan. With the increasing concertation of both forms of Van, the cell viability rapidly decreases. However, it is obvious that there is almost no difference in the effect of free Van and ApoVan in the cytotoxicity of UKF-NB-4 cells. Secondly, the time course of the treatment of UKF-NB-4 (Figure 5B) and TT (Figure 5C) cells with free Van and ApoVan was studied. A significant decrease in the UKF-NB-4 and TT cells viability is evident already after half an hour of the treatment with both the free Van and ApoVan. Again, almost no difference in the effect of free Van and ApoVan in cytotoxicity was observed. Thus, these results indicate that our assumption about the higher cytotoxicity of ApoVan due to the locally increased concentration during apoferritin-cell interaction was not met.

However, even if the encapsulation of Van in apoferritin was not successful, ApoVan may have lower side effects due to the EPR effect while maintaining the antitumor effect. This may, however, be proved only with animal experiments.

## 4. Materials and Methods

### 4.1. Chemicals and Reagents

Horse spleen apoferritin, penicillin, streptomycin and triethylamine were purchased from Sigma Aldrich, (St. Louise, MO, USA); RPMI medium, phosphate buffered saline (PBS), and fetal bovine serum (FBS) and PrestoBlueTM reagent were from Thermo Fischer Scientific (Waltham, MA, USA). Van was from LC Laboratories (St. Woburn, MA, USA), acetonitrile from J. T. Baker (Phillipsburg, NJ, USA), and Iscove’s modified Dulbecco’s medium (IMDM) from Life Technologies (Carlsbad, CA, USA). All other chemicals were purchased from Lachner (Prague, Czech Republic). The purity of all chemicals met the standards of the American Chemical Society, unless noted otherwise.

### 4.2. Preparation of Apoferritin Associated with Van 

The stock solution of Van (10 mg/mL) was prepared by dissolving Van in dimethyl sulfoxide using sonication. The effect of DMSO on apoferritin structure (denaturation) was studied by 5% native polyacrylamide gel electrophoreses (20 µg of ferritin was dissolved in three different concentrations of DMSO, incubated for 30 min; for detection, staining with ethidium bromide was performed), and it was found that there is no effect at our concentrations. Even higher concentrations may be used [39]. The encapsulation of Van into apoferritin was performed as follows: 200 μL of horse spleen apoferritin (50 mg/mL) was added to 2.9 mL of distilled water and 100 μL of Van (10 mg/mL). The pH was lowered to approx. pH 3 using 1 M hydrochloric acid and the mixture was shaken for 15 min at laboratory temperature to create a homogeneous solution. After mixing, the pH was increased to approx. pH 8 using 1 M sodium hydroxide. The mixture was mixed again for 15 min under the same conditions. The sample was diafiltered three times with 1 mL of ultra-pure water using Amicon^®^ Ultra—4 mL 3K (Merck Millipore, Billerica, MA, USA) at 9000 RCF for 10 min. 

### 4.3. Characterization of ApoVan Nanocarrier

Pure apoferritin, freshly prepared ApoVan and ApoVan prepared a week ago and stored at 4 °C (ApoVan^S^) were visually examined, and the average size of the ApoVan NPs was measured on TEM (Tecnai F20, FEI, Hillsboro, OR, USA) with the negative staining technique. The average size of Apo, ApoVan and ApoVan^S^ was also determined by DLS using Zetasizer Nano ZS (Malvern Instruments, Worcestershire, UK). Samples of apoferritin were diluted to a concentration of 10 μg/mL, placed into polystyrene latex cells and measured at a detector angle of 173°, wavelength of 633 nm and temperature of 25 °C with refractive index of dispersive phase 1.45 and 1.33 for the dispersive environment. For each measurement, Zen0040 disposable cuvettes (Brand GmbH, Wertheim, Germany) were used, containing 50 μL of the sample. the equilibration time was 120 s. Measurements were performed in hexaplicate. The surface zeta potential (ζ-potential) of the NPs normalized to 20 μg/mL of apoferritin was measured using the Zetasizer Nano ZS (Malvern Instruments, Malvern, UK). For each measurement, disposable cells (DTS1070) were employed. The number of runs varied between 20 and 40. Measurements were performed in triplicate.

Samples of ApoVan were also characterized with respect to stability. Samples were stored at 4 °C and −20 °C. During storage, aliquots were analyzed for released Van which was removed by diafiltration with distilled water using the Amicon^®^ Ultra—0.5 mL 3 K (Merck Millipore, Billerica, MA, USA) at 13,000× *g* for 15 min. The stability of ApoVan was monitored for up to 8 weeks. Released (“free Van” fraction) and retained Van (“ApoVan” fraction) were collected. Out of both fractions 25 μL was taken and mixed with 25 μL of acetonitrile. The obtained samples were analyzed using reverse phase HPLC, according to [40].

### 4.4. The Effect of pH on Release of Van from ApoVan Form 

The release of Van from ApoVan at different incubation times was determined by HPLC as previously described [40]. Samples (500 μL) of ApoVan (1.5 mM) were placed in a D-tube (molecular weight cut-off 3 kDa, D-Tube Dialyzer midi; Novagen, Darmstadt, Germany) and incubated in 14.5 mL of 0.1 M potassium phosphate buffer (pH 6.5 or 7.4) at 37 °C in the dark. At each incubation time, the sample (500 μL) of buffer with the released Van was replaced by a pure buffer and the sample was extracted twice with 1 mL of dichloromethane. The solvent was evaporated to dryness and the residues were dissolved in 50 μL of methanol before analysis.

### 4.5. Cell Culture

The difference between the effect of free Van and ApoVan on the neuroblastoma cell line (UKF-NB-4) and medullary thyroid carcinoma cell line (TT) was studied. The UKF-NB-4 cell line derived from bone marrow infiltrated by recurrent high-risk NBL, was a generous gift from Prof. Jindrich Cinatl, Jr. (University of Frankfurt, Frankfurt, Germany). The TT medullary thyroid carcinoma cell line derived from malignant cells of a patient with the sporadic form of medullary thyroid carcinoma was purchased from ATCC (Manassas, VA, USA). Cells were grown at 37 °C, 5% CO_2_ and 95% humidity in Iscove’s modified Dulbecco’s medium (IMDM) with 10% of fetal bovine serum (neuroblastoma cells) or in RPMI medium with 20% of fetal bovine serum (thyroid carcinoma cells). Both media also contained a penicillin–streptomycin mixture to prevent bacterial contamination. In the first experiment, UKF-NB-4 cells were treated with different concentrations of free Van or ApoVan for 48 h. This incubation time was chosen as it essentially corresponds to two rounds of cell division [41]. In the second experiment UKF-NB-4 and TT cells were treated with a single concentration (10 μM) of Van and/or ApoVan for a different time period of up to 48 h. At the end of each incubation period, the medium with Van or ApoVan was removed and replaced with a fresh medium. The overall cell growth was 48 h.

### 4.6. PrestoBlue Assay

The PrestoBlue fluorometric assay was used to detect cell viability in a 96-well plate format. For dose or time-response curves, cells were seeded in 100 μL of medium at a density of 1 × 10^4^ cells per well. Cells were treated with Van or ApoVan (concentration 10 μM, time of 0.5–48 h). After 48 h from seeding at 37 °C and 5% CO_2_ and 95% humidity, 5 μL PrestoBlue^TM^ was added to each well and the plate was incubated for 10 min. The fluorescence was measured using an excitation wavelength of 570 nm and emission of 610 nm by SpectraMax^®^ i3x Multi-Mode Microplate Reader (Molecular Devices, Sunnyvale, CA, USA).

### 4.7. Statistical Analysis

Data are expressed as mean values ± standard deviations. Student’s *t*-test was used for statistical comparisons.

## 5. Conclusions

Based on the characteristics obtained by analyzing the average size, the surface ζ-potential and the polydispersive index, ApoVan NPs should be stable and maintain their morphology. Although ApoVan is stable after storage at +4 °C and −20 °C for up to eight weeks, Van is gradually released into the acidic environment as well as into the physiological one (pH 7.4). Additionally, a minimal difference between the effect of Van alone and ApoVan on the UKF-NB-4 neuroblastoma cells and the medullary thyroid carcinoma (TT) cells was found. Pursuant to these results, the encapsulation of Van into the apoferritin cavity is very unlikely and Van molecules probably only bind to the surface of the protein. The suitability of ApoVan should be tested in animal experiments.

## Figures and Tables

**Table 1:** Average size, polydispersive index and ζ-potential of apoferritin nanoparticles.

	Average Size [nm] ± SD	Polydispersive Index	ζ-Potential [mV] ± SD
Apo	11.7 ± 1.1	0.361 ± 0.03	−19.6 ± 0.7
ApoVan	10.8 ± 0.9	0.512 ± 0.01	−26.9 ± 0.9
ApoVan^S^	11.7 ± 1.5	0.420 ± 0.04	−29.4 ± 1.2

## Data Availability

The data presented in this study are available on request from the corresponding author.
